# Modeling the effects of cigarette smoke extract on influenza B virus infections in mice

**DOI:** 10.3389/fimmu.2023.1083251

**Published:** 2023-03-23

**Authors:** Jerald R. Chavez, Wangyuan Yao, Harrison Dulin, Jasmine Castellanos, Duo Xu, Rong Hai

**Affiliations:** ^1^ Department of Microbiology and Plant-pathology, University of California, Riverside, Riverside, CA, United States; ^2^ Genetics, Genomics and Bioinformatics Graduate Program, University of California, Riverside, Riverside, CA, United States; ^3^ Cell, Molecular, and Developmental Biology Graduate Program, University of California, Riverside, Riverside, CA, United States

**Keywords:** influenza virus, innate immunity, cigarette smoking, influenza B virus, adaptive immunity

## Abstract

Influenza B virus (IBV) is a major respiratory viral pathogen. Due to a lack of pandemic potential for IBV, there is a lag in research on IBV pathology and immunological responses compared to IAV. Therefore, the impact of various lifestyle and environmental factors on IBV infections, such as cigarette smoking (CS), remains elusive. Despite the increased risk and severity of IAV infections with CS, limited information exists on the impact of CS on IBV infections due to the absence of suitable animal models. To this end, we developed an animal model system by pre-treating mice for two weeks with cigarette smoke extract (CSE), then infected them with IBV and monitored the resulting pathological, immunological, and virological effects. Our results reveal that the CSE treatment decreased IBV specific IgG levels yet did not change viral replication in the upper airway/the lung, and weight recovery post infection. However, higher concentrations of CSE did result in higher mortality post infection. Together, this suggests that CS induced inflammation coupled with IBV infection resulted in exacerbated disease outcome.

## Introduction

Influenza virus infections cause seasonal epidemics that result in significant disease and economic burden (1). Between 2010-2020, estimated yearly symptomatic infections caused by Influenza viruses’ range between 9-45 million cases, 140,000-710,000 hospitalizations, and between 12,000-52,000 deaths in the United States (2). Extending out to the global population, 290,000-650,000 die worldwide annually as a result of Influenza virus infections (3). Economically, these infections result in an estimated 2.8-5.0 billion dollars in medical costs in the United States alone as of 2017 (4), representing 0.014-0.03% of the US national GDP for that year (5). Therefore, to better prevent and treat influenza viral infection, it is imperative that we further examine any factors that could exacerbate disease outcomes.

Influenza viruses are negative sense, segmented, RNA enveloped viruses belonging to the Orthomyxoviridae family. There are 4 types: Influenza A virus (IAV), Influenza B virus (IBV), Influenza C virus (ICV), and Influenza D virus (IDV). Type A-C all infect humans, however IAV and IBV are primarily responsible for seasonal epidemics. Between 2000-2020, IAV remained the dominant seasonal influenza virus type in the United States (**Table 1**) (6–24). Historically, IAV has dominated research efforts and understanding of IBV has lagged behind. This gap in IBV research should be filled since IBV is also a known public health concern. For example, IBV accounted for significant percentages of known cases in the United States, as high as 45% in certain years (**Table 1**). Of the aforementioned 2.8-5 billion dollar medical cost estimate in 2017, IBV infections accounted for 37% of that total (4). Outside of the United States, IBV has achieved dominant status over IAV in Europe in some years (25). Additionally, IBV can adversely affect specific vulnerable populations. In pediatric cases for example, IBV infection can be more virulent compared to adult cases (26). Despite these sizable economic and disease burdens, IBV remains relatively understudied compared to IAV. With awareness of the impact of IBV, the field has begun to increase efforts for IBV. As evidence, both lineages of IBV have been included in seasonal Flu vaccines, dubbed the quadrivalent Flu vaccine, since 2012 in US. However, it remains largely unknown what is the impact of respiratory related lifestyle factors, such as cigarette smoking, on IBV infection, and its associated co-morbidities.

**Table 1:** Yearly IAV to IBV infection cases in the United States as Reported in the CDC MMWRs.

Flu Season	A/B Case Ratio	% IAV cases	% IBV Cases
2000-2001	5337/4625	54	46.0
2001-2002	13706/1965	87.5	12.5
2002-2003	6180/4768	56.4	43.6
2003-2004	24400/249	99	1.0
2004-2005	17750/5799	75.4	24.6
2005-2006	14355/3642	79.7	20.3
2006-2007	18817/4936	79.2	20.8
2007-2008	28263/11564	71	29.0
2008-2009	18175/9507	66	34.0
2009-2010	155591/2273	99	1.0
2010-2011	40282/13994	74	26.0
2011-2012	19285/3132	86	14.0
2012-2013	51675/21455	71	29.0
2013-2014	46727/6743	87.4	12.6
2014-2015	104,822/20,640	83.5	16.5
2015-2016	62982/28477	68.9	31.1
2016-2017	116590/45361	72	28.0
2017-2018	189716/88187	68.3	31.7
2018-2019	208153/11189	94.9	5.1
2019-2020	27617/19357	58.8	41.2

Cigarette smoking also represents a medical and environmental factor known to damage respiratory tissues. Thus, it is likely to exacerbate IBV infection and disease outcomes. Cigarette smoking (CS) results in an estimated 480,000 deaths in the United States each year, representing approximately 6.8% of the annual cigarette related deaths worldwide (27). CS is known to increase the risk and/or be causative of a number of chronic diseases, including, but not limited to: heart disease, multiple types of cancer, diabetes, and chronic obstructive pulmonary disease (COPD) (28). Smoking is also an established risk factor for infectious disease, including pulmonary bacterial infections like pneumonia (29, 30), Tuberculosis (31–34), acute respiratory tract infections in children exposed to environmental cigarette smoke (dubbed second hand smoke) (35), and viral infections like Human papillomavirus (HPV) infections (36) and Influenza A virus infection (37–39). Similarly, studies have also shown that smoking has detrimental effects on COVID-19 outcomes (40, 41).

Besides the risk, CS is also known to increase the severity of IAV disease in patients (37). Similarly, cigarette smoke has been shown to decrease weight gain (slow recovery) (42–45) and increase both lung remodeling (46) and mortality in animal models of IAV infection (43–45, 47, 48). Interestingly, multiple studies have reported that animal models of cigarette smoking do not exhibit higher viral titers than non-smoking controls post infection (43, 47–49), suggesting that worse disease outcomes are likely not due to changes in the viral replication. However, CS does appear to alter pro-inflammatory cytokine profile responses to IAV infection. Specifically, CS exposure in mice greater than two weeks appears to result in higher levels of pulmonary pro-inflammatory cytokines including (but not limited to) TNF-α, IFN-γ, IL-6, IL-12, IL-23, IL-1, IL-5, IL-10, KC, MIP-1α, IL-17, and IL-1β (44–46, 48, 50). The favorable explanation is that this increased pro-inflammatory response could give rise to exacerbation of pulmonary inflammation post infection, resulting in greater damage and slower recovery (42, 45, 46, 48, 49, 51).

Surprisingly however, to our knowledge, there is very little information regarding how cigarette smoking affects IBV infections and disease outcomes specifically. Lacking pathological, virological, and immunological profiling of smoking effects on IBV infection could result in severe lag-time between treatment development and deployment, especially in sever epidemics or situations when IBV is of particular concern. To this point, we know second hand smoke has been shown to result in not only higher incidents of infection, but also hospitalization in infants and children (52–54), and because IBV infection can be severe in children, it is critical we further investigate the role of CS in IBV infection. To this end, it is critical to establish an experimental model of how cigarette smoke affects the pathology, virology, immunology, and disease outcomes from IBV infection in mice.

Here, we developed an animal model system to better understand how aspects of CS may affect IBV infections by treating mice for two weeks with liquid cigarette smoke extract (CSE), then infecting them with IBV. Our results showed that exposure to CSE decreased IBV specific antibodies but oddly did not compromise their neutralization potency for IBV. Similar to previous studies in IAV, we also did not observe an impact of CSE on virus replication, and associated disease outcomes. Intriguingly, we observed about a 2-fold increase in IBV specific activated splenocytes from animal exposed to CSE versus the control animals. Additionally, we observed a dose dependent effect of increasing concentrations of CSE on mortality in mice. These data represent the first information regarding the pathological and immunological effects of water-soluble components of CS on IBV infection *in vivo* and suggested that there is a negative impact on IBV disease outcome. Our studies provide an experimental platform to further dissect the impact of CSE on IBV infection.

## Materials and Methods

### Virus and cells

Influenza B/Victoria/2/87 virus was propagated in pathogen free eggs purchased from Charles River laboratories Inc. and stored at -80°C. A549 and Madin-Darby canine kidney (MDCK) cells were cultured at 37°C in DMEM medium supplemented with 10% FBS, or MEM medium supplemented with 10% FBS, respectively.

### Cytotoxicity assay

To evaluate the impact of CSE on cell viability, we used the Cell Counting Kit-8 (CCK-8), Dojindo Inc. Briefly, A549 cells were plated at 2.75x10^5^ cells per well in 96 well plates in DMEM supplemented with 10% FBS for 24 hours (hrs). Cells were subsequently exposed to varying concentrations of CSE, ranging from 40X to 0.16X, with a 3-fold dilution, and maintained at 37°C for an additional 24 hours. CCK-8 solution (10 μL per well) was added, followed by an additional incubation for 2 h. The absorbance was measured at 450 nm.

### Multi-step growth curve

To evaluate viral replication under the influence of CSE, A549 cells were plated at 3x10^5^ cells per well in 6 well plates in DMEM supplemented with 10% FBS for 24 hrs. Medium was aspirated, then cells were treated overnight with either PBS, 1x CSE, or 2.5x CSE diluted in DMEM with 10% FBS. The following day, media with CSE or PBS was removed, and cells were infected with a multiplicity of infection (MOI) of 0.05 of Influenza B/Victoria/2/87 virus diluted in PBS/BSA/PS (1x PBS, 0.42% BSA, 100ug/ml Pen-strep, 0.8mM CaCl_2_-2*H_2_O, 1mM MgCl_2_-6H_2_O) and incubated at 33°C for one hour. Then, virus solutions were aspirated and replaced with 1ml of post infection media (1x DMEM, 0.35% BSA, 100U/ml Pen-strep, 2mM L-glutamine, 0.15% sodium bicarbonate, 20mM HEPES pH 7.0, 0.25ug/ml TPCK). Infection samples were collected at 24 and 48 hours post infection. The virus concentrations were evaluated by standard plaque assays.

### Plaque assay

MDCK cells were plated in 12 well plates at 5x10^5^ cells/well the night before in MEM supplemented with 10% FBS. Virus was serially diluted in PBS/BSA/PS. MEM media from cells was aspirated and replaced with 200µl of virus dilution for 1hr at 33°C. Plates were rocked every 15 min. Virus was aspirated and replaced with plaque overlay (1x EMEM, 0.21% BSA, 100µg/ml Pen/Strep, 2mM L-Glutamine, 0.22% Sodium Bicarbonate, 10mM HEPEs pH 7.0, 0.1% D-dextrose, 0.7% Avicel, 1µg/ml TPCK). Plates were incubated at 33°C for 72hrs. Cells were fixed with 3.7% Formaldehyde in 1x PBS for 1hr, then stained with 0.08% Crystal Violet.

### Mice

6-8 weeks old Female BALB/cJ mice were purchased from the Jackson Laboratory and housed in a pathogen free vivarium facility at the University of California, Riverside. Food and water were available ad libitum.

### Cigarette smoke extract exposure

Cigarette smoke extract was prepared as previously described (55, 56). Briefly, cigarette smoke from 40 commercially available Marlboro Class A Cigarettes were filtered through 12.5ml of sterile 1xPBS at a rate of 1 cigarette every 1 minutes in a chemical hood. Cigarettes were smoked until they reached the filter, then replaced. The resulting liquid was filter sterilized through a 0.22uM filter and classified as “40X cigarette smoke extract (CSE)”. 40x CSE was aliquoted and frozen at -80°C until use.

6 to 8-week-old BALB/cJ female mice were anesthetized with isoflurane, then intranasally inoculated with 50μl of specified concentration of CSE (diluted in sterile PBS) or PBS as a mock control. Mice were daily treated in the same manner, 6-days per week for two weeks.

### Influenza virus infections

After two weeks of CSE exposure, mice were isoflurane anesthetized and intranasally inoculated with 50μl of Influenza B/Victoria/2/87 WT virus diluted in PBS/BSA/PS. Total PFU per mouse given were as specified in figures. Mice were sacrificed on day 0, 3, 6, or 21 post infection depending on the experiment.

### Lung pathology

After two weeks of CSE or PBS treatments, mice were infected with 10^5^ PFU B/Victoria/2/87 WT virus per mouse. Mice were sacrificed 0 and 3 days post infection, and lungs were extracted, washed in 1x PBS, then fixed in 4% formaldehyde at room temperature. Lungs were dehydrated, embedded in paraffin, and lung sections were subjected to Hematoxylin and Eosin (H&E) staining.

### Hematoxylin and eosin staining

Mice were euthanized with CO_2_, and lungs were extracted and washed with PBS, the fixed it with 4% formaldehyde for 72 hrs at room temperature. Lungs were subsequently dehydrated with 70%, 80%, 90%, and 95% ethanol for 2, 2, 1, and 1hr respectively, then dehydrated again with 100% ethanol for 1 hr. After xylene treatment, lungs were immersed in liquid paraffin wax. Lungs were sectioned using microtome (Lecia Microsystems, Leica RM2235), at approximately 4μm thickness per slice. The slices were then attached to a glass slide and dried at 45°C for 12 hrs. Last, slides were Hematoxylin-Eosin stained, dried, fixed with neutral resin, then covered with cover slips.

### BAL fluid collection

21 days post IBV infection, mice were sacrificed. Tracheas were exposed and incisions were made above the manubrium. One ml of sterile PBS was pushed through the incision and out the nasal cavity for collection. BAL fluid was clarified by centrifugation, aliquoted, and frozen at -80°C until analysis.

### Enzyme-linked immunosorbent assay for IgG and IgA

To assess the levels of virus-specific IgG and IgA antibodies present in samples from IBV infected mice, ELISAs were performed on blood sera (for IgG) or lavage fluid (for IgA) samples. In brief, 96 well MaxiSorp ELISA plates (Thermo Fisher Scientific, #442404, Rochester, NY) were coated with 50µl of 10µg/ml purified B/Victoria/2/87 WT virions. Wells were blocked at room temperature with PBS containing 1% dried milk and 0.1% Tween 20 (blocking buffer) for 2hrs, washed with PBS containing 0.1% Tween 20 (wash buffer), and subsequently incubated with blood sera or lavage samples serially diluted in blocking buffer. After 2hr room-temp incubations, plates were washed with wash buffer and incubated with secondary horse radish peroxidase conjugated antibody (Southern Biotech #1040-05 for IgA; Millipore, CAT# AP503P, Temecula, Ca for IgG) for 30min at room temperature. Plates were washed with wash buffer and incubated with colorimetric substrate (o-phenylenediamine dihydrochloride, Invitrogen, Carlsbad, CA) for 30min at room temperature, then read with a plate reader measuring optical density at 450 nm (OD_450_).

### IFN-γ evaluation

Mice were sacrificed 6 days post IBV infection. Spleens were removed and washed in 5ml of R10 media (RPMI media supplemented with 2mM L-glutamine, 100ug/ml Pen-strep, 100mM Hepes pH 7.0, and 10% FBS). Spleens were homogenized through a 40 µM cell strainer, washed with 5 ml of R10 media, centrifuged at 1000g for 5 min, then aspirated. Homogenates were treated with 3ml of Ammonium-Chloride-Potassium (ACK) lysis buffer (NH_4_Cl 150mM, KHCO_3_ 10mM, EDTA 0.1mM, pH to 7.2) for 10min and neutralized with 10ml of R10 media. Homogenates were centrifuged, aspirated, resuspended in 4ml R10 media, then counted. 3x10^5^ cells/well were plated in triplicate per spleen in 96 well plates in R10 media. Boiled B/Victoria/2/87 WT virus was added to a final concentration of 30ug/ml for stimulation, and plates were placed at 37°C for 72 hours. Anti CD3/CD28 antibody at 20ug/ml and R10 media was used as positive and negative controls respectively. Supernatants were harvested, clarified by centrifugation, then frozen at -80°C until ELISA analysis.

We used ELISAs to evaluate IFN-γ content in the supernatant samples. Specifically, Nunc Maxisorp plates were coated with 50µl of 0.5ng/µl Anti-mouse IFN-γ purified antibody (Invitrogen eBioscience #14-7313-85) overnight at 4°C. Wells were washed 3x with wash buffer (PBS with 0.05% Tween 20). 50 µl of supernatant samples were diluted 1:10 in dilutant buffer (PBS with 1% BSA and 0.05% tween 20) and added to wells for 2 hours at 37°C. Wells were washed, then treated with 50µl (0.5 µg/ml) of biotin conjugated anti-mouse IFN-γ antibody (Invitrogen ebioscience #13-7312-85) for 1hr at 37°C. Wells were washed, then treated with 100 µl (0.5µg/ml) of HRP conjugated streptavidin (Jackson Immunoresearch #016-030-084) for 30min at 37°C. Wells were washed, then incubated with colorimetric substrate (o-phenylenediamine dihydrochloride, Invitrogen, Carlsbad, CA) for 30min and read with plate reader measuring optical density at 450 nm (OD_450_).

### Microneutralization assay

To assess neutralizing potency of antibodies against the challenge virus, we performed microneutralization assays. Briefly, 6x10^4^ MDCK cells were plated in 96 well plates. 24hr after plating, 2000 PFU of B/Victoria/2/87 WT virus was incubated with serum samples serially diluted in PBS containing 0.35% BSA for 1hr at 33°C. Virus-serum mixtures (100μl) were added to MDCK cells (MOI=0.003) and incubated at 33°C for 1hr, then washed with PBS. Cells were then incubated overnight at 33°C in MEM media containing 0.35% BSA, 2mM L-glutamine, 0.15% NaHCO_3_, and 2 mM HEPES pH 7.0, and 1µg/ml TPCK. 24 hours post infection (hpi), cells were fixed with 100% methanol for 20min at -20°C and washed with PBS. Cells were blocked at room-temp with PBS containing 1% dried milk and 0.1% Tween 20 (blocking buffer) for 1hr, and then incubated with sera from B/Victoria/2/87 infected mice diluted in blocking buffer. After 1hr room-temp incubations, plates were washed with wash buffer and incubated with secondary anti-mouse horse radish peroxidase conjugated antibody HRP (Millipore, Temecula, CA) IgGγ for 30min at room-temp. Plates were washed and then incubated with colorimetric substrate (o-phenylenediamine dihydrochloride, Invitrogen, Carlsbad, CA) for 30min and read with plate reader measuring optical density at 450 nm (OD_450_).

### RNA extraction and qRT-PCR

Mice were euthanized 3 days post infection by CO_2_ and lungs were immediately extracted and placed in 1ml of Trizol reagent (Thermo Fisher Scientific). Samples were homogenized, then frozen at -80°C until time of RNA extraction. 250µl of Chloroform was added. Samples were vortexed and centrifuged at 20,000g for 15min at 4°C. The RNA from the aqueous phase was precipitated with isopropyl alcohol at a ratio of 1:1.1 using glycogen as a carrier. The resulting RNA pellet was washed with 70% ethanol, air dried, and resuspended in nuclease-free water.

To remove contaminating genomic DNA, RNA was treated with DNAse I (Ambion #2222, Austin, TX). DNAse was removed by phenol/chloroform extraction and RNA was resuspended in nuclease free water. cDNA was synthesized from 1ug of RNA per sample using Superscript II in 20µl reactions (18064-022, Invitrogen, Carlsbad CA). qRT-PCR reactions used 2µl of a 1:10 dilution of cDNA, 400 nM of each primer, and 10µl of 2x Radiant Green Lo-Rox qPCR mix (QS1005, Alkali Scientific, Fort Lauderdale FL). β-Actin internal control was used to normalize results.

### Statistical analysis

The experimental data were analyzed by the student *t*-test or the two-way ANOVA depending on the specific setting using the GraphPad Prism V. 9.0.

### Ethics and biosafety statement

Animal studies were approved by University of California, Riverside Institutional Animal Care and Use Committee (IACUC) and performed in the biosafety level 2 facility. All animals were cared for in the Animal Resources Facility under specific-pathogen-free conditions in appliance with the Institute for Laboratory Animal Research Guide for the Care and Use of Laboratory Animals, 8th edition.

## Results

### CSE suppressed IBV replication in A549 cells

**Figure 1:**
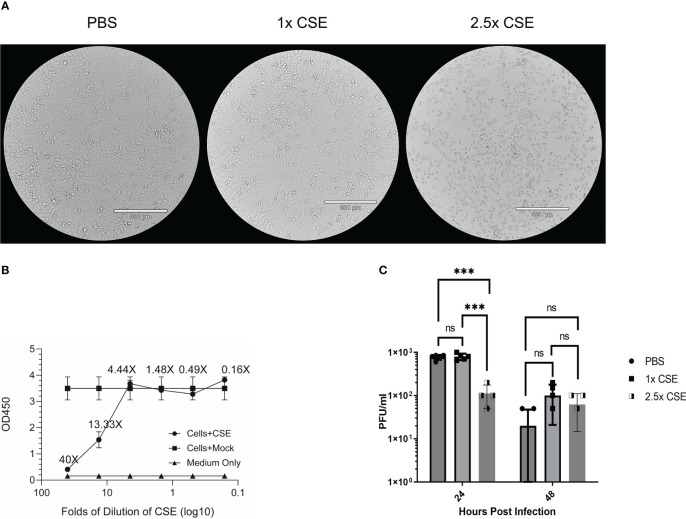
CSE suppressed viral replication ex vivo. A549 cells were treated for 24hrs with either PBS (mock), 1x CSE, or 2.5x CSE **(A)**. Cytotoxicity of CSE on A549 cells were further analyzed with CCK-8 kit **(B)**. Cells were infected with Influenza B/Victoria/2/87 at an MOI = 0.05. Supernatant samples were taken at 24 and 48 hpi and tittered by standard plaque assay **(C)**. A standard 2-way ANOVA with multiple comparisons was used for statistical analysis in PRISM software 9.0, *** = *p*<0.0001. N=4 per treatment group. NS, Not statistically significant.

Duffney et al. has previously shown that there was more WSN (A/WS/1933 H1N1) IAV infectivity in human airway epithelial cells exposed to cigarette smoke compared to the control cells (57). To evaluate whether there is a similar impact in human lung cells exposed to the water-soluble components of CS on IBV infection, we treated A549 cells with either PBS (mock), 1x CSE, or 2.5x CSE for 24hr, then infected with Influenza B/Victoria/2/87. We noted that 24 hours post CSE treatment, 1x CSE and mock control cells appeared to have similar morphology (**Figure 1A**). However, 2.5x CSE treated cells appeared to cease proliferation, likely due to toxicity from high dose CSE. Yet, these cells were still attached to the plate (**Figure 1A**). To more quantitively evaluate the cytotoxicity of CSE, we measured cell viability using the CCK-8 kit (**Figure 1B**). The results showed similar readings between cells with or without CSE treatment at 4.44X and below. This suggested that concentrations of CSE at 4.44X and below exhibited no apparent negative impact on cell viability. Post infection, 1x CSE did not appear to increase or decrease virus replication, but 2.5x CSE did appear to significantly decrease viral titers 24 hours post infection (hpi) (**Figure 1C**).

### Low dose of CSE did not exacerbate IBV infection

**Figure 2:**
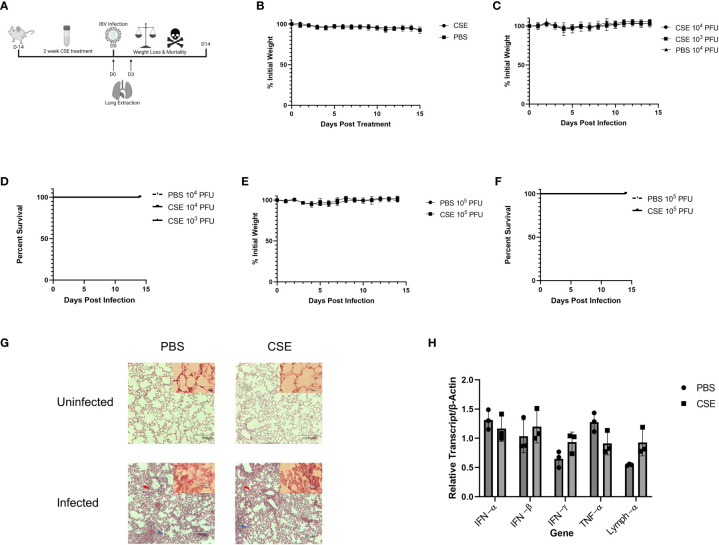
1X CSE treatment does not affect mice weight loss or survival before or after IBV infection. **(A)** 6–8-week-old female BALB/cJ mice were treated intranasally with 50µl of 1xCSE daily, six days per week, for two weeks total. Weights of mice were monitored during 1x CSE treatment **(B)** and after infection **(C)** with 10^3^, 10^4^, or 10^5^ **(E)** PFU/mouse. Survival was monitored up to 14 days post infection for **(D)** 10^3^/10^4^ or 10^5^ **(F)** PFU/mouse groups. N=5 for all groups. Lungs were harvested from 1X CSE treated mice at the day of infection, Day 0, or three days post IBV infections. For mice of 3 DPI, half of tissues were fixed for H&E staining analysis **(G)** and the rest were used for qPCR gene expression analysis of pro-inflammatory molecules **(H)**. Larger lung pictures are 10X magnification, while smaller picture in upper right corner of lung histology represents 20x magnification. Red arrows indicated thickening of the alveolar septa with congestion, blue arrows indicate the infiltration of inflammatory cells. With higher resolution at 20X, a large number of neutrophils and lymphocytes were only present in infected samples. Statistical significance for figure were determined by 2-way ANOVA with multiple comparisons.

To examine the pathological effects of cigarette smoke on IBV infection *in vivo*, we intranasally inoculated 6-8 week old female BALB/cJ mice with 1x CSE for two weeks, 6 days/week (**Figure 2A**). For this period of treatment, 1x CSE exposure did not affect the weights over a two-week period (**Figure 2B**). Furthermore, 1x CSE exposure did not substantially increase pathological damage in the lungs of mice compared to PBS control mice (**Figure 2G**, Top). Subsequently, we infected these mice with 1X10^3^, 1X10^4^, or 1X10^5^ PFU/mouse of IBV (Influenza B/Victoria/2/87). We observed mice body weight changes for 14 days post infection. We found that 1x CSE exposure did not increase weight loss during this two-week period post infection, regardless of the dose of IBV compared to PBS control mice (**Figures 2C, E**), nor did 1x CSE exposure have any effect on mortality among different groups of mice (**Figures 2D, F**). Finally, lung histology on tissue from three days post infection indicated immunocyte infiltration only in infected samples with or without CSE treatment. However, the phenomenon was not observed in samples from CSE treatment alone (**Figure 2G**, Bottom). This suggests that our current CSE dose is not high enough to exhibit a significant negative impact on disease outcomes.

**Figure 3:**
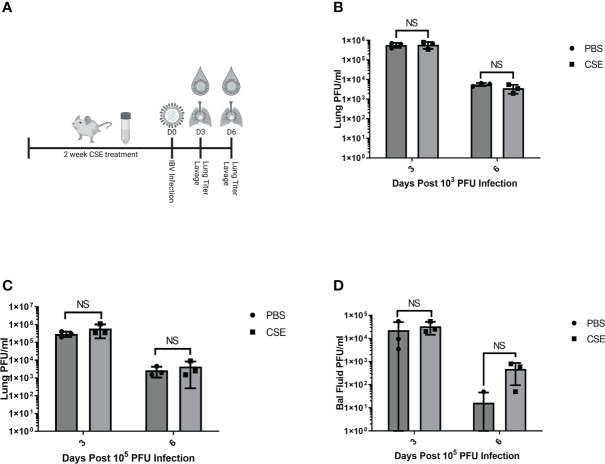
1X CSE treatment does not affect respiratory viral replication or pathological responses. **(A)** schematic describing 6–8-week-old female BALB/cJ mice treated with 1X CSE and infected with IBV. Virus lung replication was measured on day 3 and day 6 post 1x10^3^ **(B)** or 1x10^5^ PFU/mouse of IBV infection **(C)**. **(D)** Virus replication was also measured from upper airway lavage fluid collected from 1x10^3^ PFU infected mice by standard plaque assay. Significance was determined by standard students *t*-test. N=3. NS, Not statistically significant.

We next assessed the potential impact of CSE on the viral pulmonary replication and immunological responses post IBV infection. We treated mice with 1x CSE and infected as described in earlier sections (**Figure 3A**). We observed that with both low and high doses of IBV, 1x CSE exposure did not affect the amount of virus detected in the lungs from mice at 3 and 6 days post infection (dpi) (**Figures 3B, C**) compared to control PBS groups. Similarly, we did not find any difference between 1x CSE and PBS viral titers in the upper respiratory fluid 3 or 6 dpi (**Figure 3D**). Also, we found that 1x CSE treatment did not have a significant impact on pro-inflammatory cytokine gene expression 3 dpi (**Figure 2H**).

**Figure 4:**
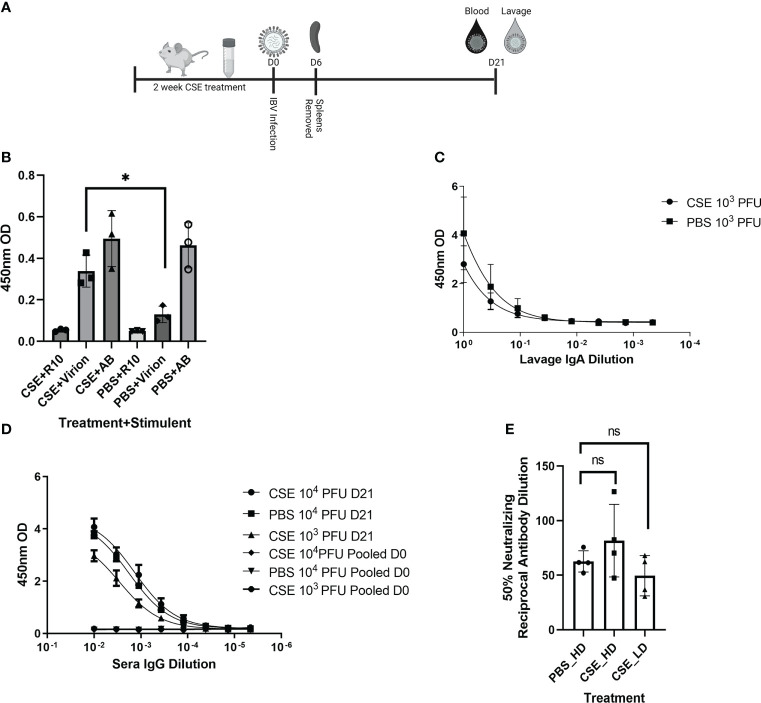
1X CSE treated mice do not exhibit altered adaptive immune responses post IBV infection. As shown in schematic **(A)** mice were treated with 1x CSE for two weeks and infected with either 10^3^ or 10^4^ PFU/mouse of IBV. Spleens from 10^3^ PFU infection group were removed 6 DPI, homogenized and stimulated with either IBV virion, R10 media only, or Positive Control Antibody CD3/CD28 (AB). **(B)** IFN-γ expression was measured from stimulated splenocytes by ELISA. N=3. **(C)** Upper airway lavage fluid was used to measure IgA antibody titers 21 DPI by ELISA from mice infected with 10^3^ PFU of IBV **(D)** Blood sera was drawn from mice infected with 10^3^ or 10^4^ PFU of IBV 21 DPI to measure IBV specific IgG responses by ELISA (N=5) or **(E)** neutralizing antibody titers as calculated from the 50% Reciprocal Inhibition Titer. Two-way ANOVA was used to test significant differences with multiple comparisons, * indicating *p*<0.5. NS, Not statistically significant.

**Figure 5:**
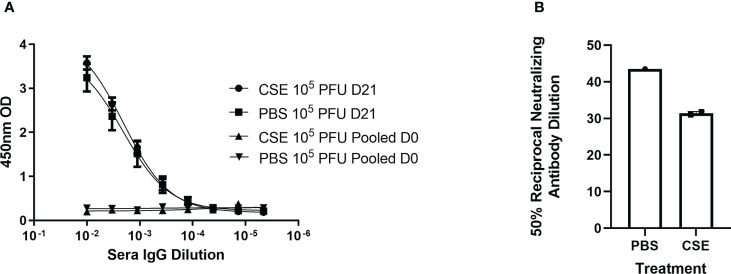
1x CSE treatment does not affect IgG or neutralizing titers of mice infected with higher doses of IBV. Mice were treated with 1x CSE as in **Figure 4**, and infected with 10^5^ PFU/mouse of IBV. **(A)** Blood sera was drawn from mice infected with 10^3^ PFU (LD) or 10^4^ PFU (HD) of IBV 21 DPI to measure IgG responses by ELISA (N=5) or **(B)** neutralizing antibody titers as calculated from the 50% Reciprocal Inhibition Titer. Two-way ANOVA was used to test significant differences.

Because smoking has been shown to alter innate and adaptive immune responses post IAV infection in some reports (44–46, 58), we went further to determine whether CSE exposure influences the host immune responses after IBV infection. Here, we examined both cellular and humoral responses through evaluating IFN-γ production from the IBV specific splenocytes, IBV specific IgA level from nasal lavage samples, and IBV specific IgG levels from sera samples (**Figure 4A**). Even though we observed significantly higher IFN-γ production from splenocytes of CSE mice versus those of PBS control animals (**Figure 4B**), we did not observe a discernable difference in PBS *vs* CSE treated animal in their IgA (**Figure 4C**) or IgG (**Figure 4D**) titers at 21dpi. Furthermore, we evaluated the potency of those IBV specific IgGs by microneutralization assays. Similarly, we did not detect significant difference between CSE or PBS treatment groups (**Figure 4E**). To evaluate whether our observation is independent of IBV dose usage, we repeated the experiments with a higher dose infection at 1x10^5^ PFU/mouse. Similarly, we found that neither IgG (**Figure 5A**) nor neutralization titers (**Figure 5B**) differed between CSE or PBS groups. Collectively, our results suggest that early cellular immune responses are elevated in CSE mice, but mucosal and humoral immunity by later stages post infection have equalized. However, this is likely due to the low dose of CSE used here in these studies. At three days post infection, lung histology indicated cell infiltration only in infected samples regardless of CSE treatment, which was not observed in samples from CSE treatment alone (**Figure 2G**, Bottom). This suggests a likely caveat that our current CSE dose is not high enough to impact on host immune responses.

### Increasing concentration of CSE reduces survival of mice post IBV infection

**Figure 6:**
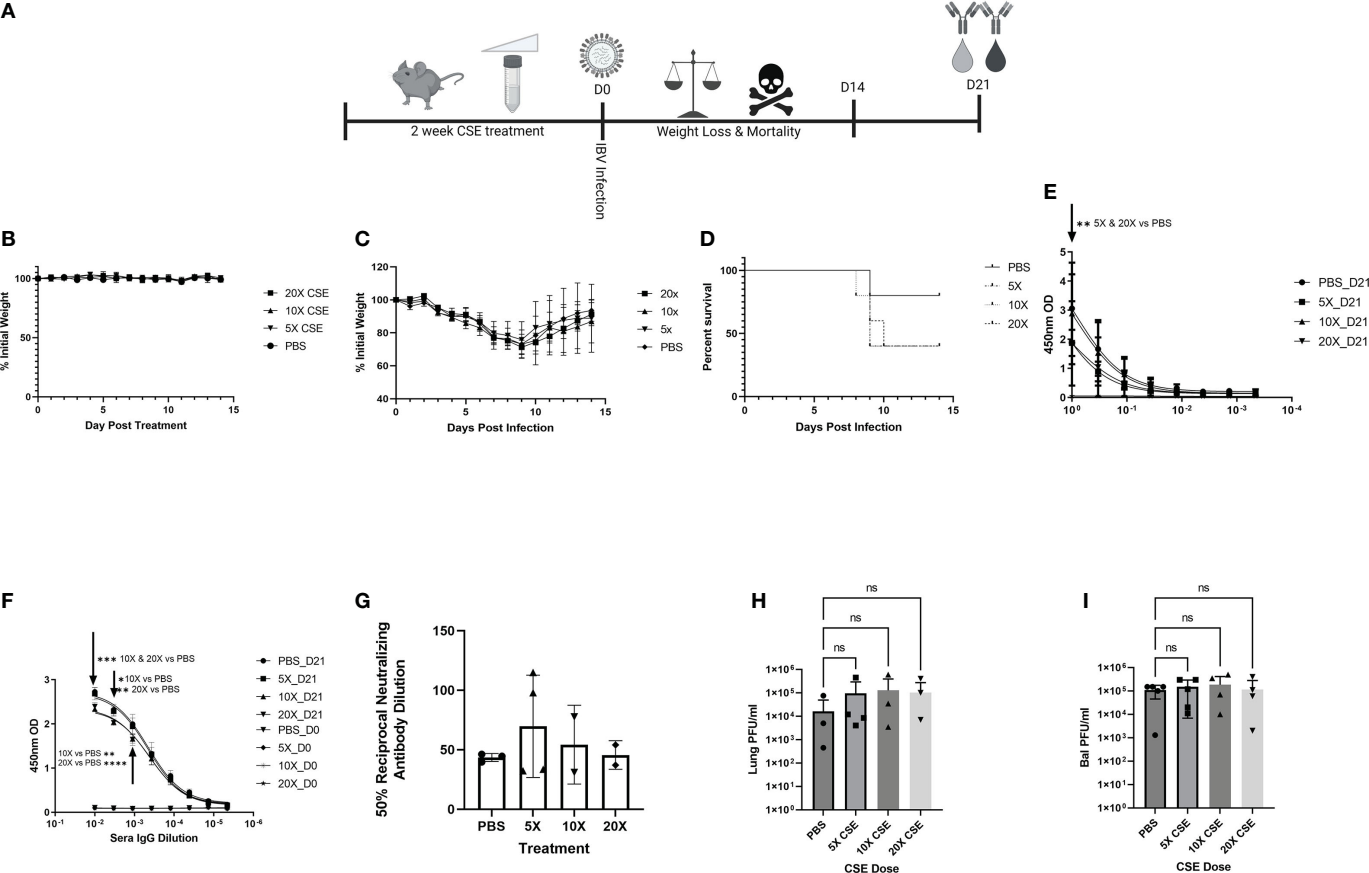
Increasing concentrations of CSE reduced survival. **(A)** Schematic showing 6-8-week-old female BALB/cJ mice were treated intranasally with 50µl of CSE, ranging from 5x to 20x, daily for six days per week, for two weeks total. **(B)** Weights of mice were monitored during CSE treatment and **(C)** after infection with 10^5^ PFU/mouse of IBV for 14 days. **(D)** Survival was monitored for up to 14 days post infection, N=5 for all groups. **(E)** Lavage IgA or **(F)** Sera IgG specific for IBV from samples collected at 21 DPI was determine by ELISA from surviving mice, and **(G)** neutralizing antibody titers were calculating from microneutralization assays. Two-way ANOVA was used to test significant differences. In a second experiment, 5 mice per group were CSE treated and infected as in schematic 6A, and viral replication for the lungs **(H)** and upper bal fluid **(I)** was measured with standard plaque assay 3 DPI. One-way ANOVA with multiple comparisons was used to test significant differences to PBS group. NS, Not statistically significant. * = p=0.01, ** = p<0.0077, *** = p=0.0001, **** = p<0.0001.

Smoking commonly varies among people, typically between 1 cigarette to multiple packs a day (https://www.lung.org/research/trends-in-lung-disease/tobacco-trends-brief/overall-tobacco-trends). To better mimic the physiologic condition, but more importantly to mimic the heavy smoking conditions, we further tested higher dose of CSE on IBV pathology, disease outcome, and immune responses. To this end, we first treated mice as described in **Figure 6A** with increasing amount of CSE. We observed that mice exhibited similar weight changes among different groups during the two-week CSE treatment period. The result suggested that increasing concentrations of CSE did not have an overwhelming impact on mice with up to 14 days treatment resulting in no significant effect of weight changes (**Figure 6B**). Following the same amount (10^5^ PFU) of IBV infection, based on the weight records for surviving animals, we did not find significant differences in weight between our CSE treatment groups and the PBS mock treatment group (**Figure 6C**). However, from our survival data, we observed that the survival rate was inversely correlated with the amount of CSE used (**Figure 6D**). To further assess impact of high dose CSE on humoral responses, we tested IgA levels of nasal wash samples (mucosal) and IgG level of sera samples (systematic). Intriguingly, we observed a significant decrease in IBV specific IgA titers only for the undiluted samples (**Figure 6E**) and a more profound significant decrease in IBV specific IgG titers up to around 900-fold of dilutions of original sera (**Figure 6F**). On the contrary, we did not observe a difference in IgG neutralizing titers for IBV (**Figure 6G**) between CSE treated animals and PBS controlled animals. Furthermore, with increasing amounts of CSE used, we observed a decrease in survival following the subsequent IBV infection. Finally, we treated mice with CSE, subsequently infected them as in **Figure 6A** and assessed the potential impact of increasing CSE concentrations on the viral pulmonary and upper bal fluid replication 3 DPI (**Figures 6H, I**). Similar to 1xCSE treatments, increasing concentrations of CSE did not significantly alter viral replication in the lungs or upper bal fluid. Together, these facts revealed the CSE did not have direct negative impact on viral replication outcomes, for both upper and lower respiratory tracts.

Overall, we established a smoking model system for IBV using water-soluble components of CS. We found that the treatment negatively affected IBV infection outcomes and dampened host immune responses. The results validate that our smoking system recapitulated the disease outcomes of natural smoking behavior upon viral infection. Together, we provided a valuable resource to understand the impact of CS on IBV infection.

## Discussion

Cigarette smoking increases the risk of IAV infection and exacerbates negative health outcomes, increasing both the time to recover and mortality. However, there is very little data on how cigarette smoking affects IBV infection, disease and to what degree. To this end, we developed an *in vivo* smoking model to study the pathological, immunological, and viral effects cigarette smoking may have on IBV infections. This was accomplished by pre-treating mice for two weeks with various concentrations of CSE, then infecting them with IBV and monitoring morbidity, mortality, lung inflammation, viral pulmonary and upper airway replication, and IBV specific serum and mucosal antibody levels. Ex vivo, IBV viral replication is not altered by 2.5x CSE treatment in A549 cells. *In vivo*, weight loss and mortality post IBV infection were not affected by 1x CSE regardless of the IBV dose compared to PBS control mice. Similarly, IgA, IgG, and neutralizing IgG levels were all similar in 1x CSE and PBS mock controls. However, 1x CSE induced a roughly 2-fold increase in IBV specific spleenocyte IFN-γ levels compared to PBS controls. Finally, increasing concentrations of CSE resulted in increased mortality compared to PBS controls after subsequent IBV infection, a significant decrease in IBV specific IgA or IgG levels but did not impact weight loss. Together, our system established a platform for further study of CS on IBV and provided first *in vivo* data on impact of CS on IBV infection in model systems.

Studying cigarette smoking and determining the specific chemical or compound in CS responsible for certain pathological or immunological responses is difficult for many reasons. Noah et al. measured live IBV vaccine RNA and specific cytokine levels post vaccination in nasal lavage fluid from active young smokers, secondhand smoke exposed, and never smoker groups (59). They noted that smokers had higher levels of IBV vaccine RNA and lower IL-6/IFN-γ levels compared to never smoker controls. Noteworthy, their conclusions were heavily influenced by variation in daily cigarettes consumed, type of cigarettes smoked, age, genetic background, unknown co-morbidities, other environmental factors, and use of attenuated vaccine virus.

To minimize the impact from those factors, it is necessary to perform a similar evaluation in a better controlled experimental system. Humans are exposed to at least 3 different types of smoke from cigarettes: firsthand, secondhand, and thirdhand smoke. Firsthand smoke comes from a person inhaling smoke through the cigarette directly into their lungs, also known as “mainstream” smoke (60), while secondhand smoke is smoke released into the environment from the lite side of the cigarette or from exhaled smoke and can be unintentionally inhaled by bystanders, a form of “side-stream” smoke (61). Thirdhand smoke come from either first or secondhand smoke that has settled or built up on furniture or surroundings that subsequently come into contact with people interacting with the smoke covered objects (62). Many systems that model cigarette smoking in mice place the mice in plexiglass chambers and expose them to mixtures of air and cigarette smoke pumped into the chamber for a specified time (whole body exposure), simulating side-stream smoke exposure or utilize nose only exposure systems (63), creating difficulty in both replicating CS exposure at relative human mass to cigarette ratios and study to study experiments.

Traditionally, the experimental system is built on the usage of a smoking chamber. Even though it can better mimic natural respiratory conditions, it suffers from the imprecise inoculation amount, let alone the financial requirements necessary for purchase. Here, we established a system based on the usage of water-soluble components from CS. CS is comprised of over 7000 chemicals and compounds. Our system will allow us to quickly distinguish water soluble component effects of cigarette smoke on IBV infection from the non-water soluble effects with fewer confounding factors. Additionally, it is superior in financial cost and prevents research personnel from handling mice that otherwise may be covered in toxic or carcinogenic components of cigarette smoke collected on their fur from side-stream smoke exposure. All these factors make this system a simple yet robust platform for evaluating CSE on respiratory viral infection.

We found that CSE treatment did not affect weight loss at any concentration from 1x to 20x. This is curious as smoking has been shown to result in weight loss in mice (64, 65). At least three factors could partially explain this lack of weight loss: a) the CSE we made contains only the water-soluble components of cigarette smoke, b) the mice were not exposed to CSE long enough to induce physical changes, or c) there were chemical variation in the cigarettes we used compared to previous studies. During the actual act of smoking cigarettes, there would be constant exposure of the lungs to water soluble and insoluble components of CS. To make our CSE, we bubble CS through PBS to capture the water soluble components, but allow the rest of the smoke to escape through the pump. Subsequently, any water insoluble particles that are trapped on the liquid surface are mostly removed by filter sterilization. As such, it’s possible the water insoluble particles or the combination with soluble components are necessary to induce weight loss. For CSE exposure length, previous studies have shown that there is a difference in pro-inflammatory cytokine response profiles depending on CS exposure of less than or greater than 2 weeks (66). It is possible that CSE exposure more than 2 weeks could have yielded a more significant effect on morbidity and mortality. Animal model studies with IAV range from as few as 3 days (67) to as long as 6 months (50). Given that there is huge variation in treatment period and amount of cigarettes used, it is not surprising to observe no significant weight loss from CSE treatment alone. Additionally, the brand of cigarette used in a study may have potential consequences on disease outcomes, including damage to the lungs. For example, Goel et al. found that among 27 brands of US commercially available cigarettes, there was as much as a 12-fold variation in free radicals in the gas phase of the CS (68). These free radicals can cause damage to cellular membranes and DNA (69), resulting in tissue damage to exposed organs. Because cigarette smoke contains over 7000 different chemicals and compounds (70), variation in which cigarettes are used in academic studies are likely going to lead to phenotypic variation post infection. Nevertheless, our CSE treatment did exhibit negative impacts on experimental animals, which resulted in decreased survival after subsequent IBV infection in a CSE dose dependent manner. The difference in weight loss warrants necessity for future studies to further titrate the specific amount, treatment time and types of CS or CSE.

Our data indicates that 1x CSE treatment did not impact IBV viral loads at 3 or 6 dpi with high or low doses of IBV or CSE. Gualano et al. has reported that cigarette smoke exposure in mice can lead to a moderate increase to viral loads (42). However, more reports indicate CS exposure does not impact viral loads of IAV infections, which is in line with our findings (43, 47–49). This would suggest that worse disease outcomes in our smoking model is likely not due to increased viral burden. Our speculation is in line with previous reports, which have correlated the final severity of disease outcome with the elevated inflammatory responses post IAV infection in smoking conditions, rather than with viral replication.

We noted interestingly that 1x CSE treatment resulted in increased IFN-γ production in spleenocytes compared to the PBS controls. IFN-γ promotes differentiation and proliferation of CD8^+^ T-cells and upregulates antigen presenting cell MHC II expression, aiding in CD4^+^ T-cell activation (71, 72). Only a specific set of immune cells produce IFN-γ, including CD8^+^ cytotoxic T-cells, B cells, and antigen presenting cells (APCs) (73). Our data suggests that post infection, CSE treatment may have resulted in either an expansion of IBV specific immune cells or the spleenocyte immune cells are producing more IFN-γ than none CSE treatment under the same IBV specific stimulation. This was also mentioned earlier that there was a time dependent effect of CS on immune cytokine responses to infection (66). It is possible that elevated IFN-γ responses post infection could reflect higher inflammatory responses (attracting more cells) post IBV infection in 1x CSE treated mice. Interestingly, this differs from IAV data, as Feng et al. has shown that there was reduced IFN-γ from the lungs of CS mice, as well as reduced numbers of IFN-γ^+^ cells from lungs and spleens of CS treated mice compared to control mice (43). It is possible this represents a potential pathological difference between IAV and IBV models of smoking, but it also may likely reflect experimental parameter differences including 1) exposure time, 6 weeks CS exposure *vs* 2 weeks CSE exposure and 2) exposure materials, CS versus CSE. Additionally, treatments with higher concentrations of CSE led to higher mortality after IBV infection. This suggests that higher concentrations of CSE are resulting in higher levels of inflammation post infection and could be responsible for exacerbating disease outcomes. Indeed, a number of IAV/CS studies have found higher lung and upper airway cell infiltration in CS mice compared to control groups (46, 48, 49).

Together, our results show that our system is a valid, rapid, and safer method to explore the effects of CS on IBV pathology and immune response compared to traditional experimental chamber models. We used the system to provide evidence to validate the negative impact of smoking on IBV infection. Together, we provided a valuable resource to understand the impact of CS on IBV vaccination and coinfections from different respiratory viruses, including IAV and even SARS-CoV-2. Furthermore, our model can serve as a platform for initial testing of the efficacy of anti-viral drugs and treatments under smoking conditions or could be used to examine if or how treatments designed to alleviate smoking consequences like inflammation affect infection outcomes. In result, these studies would provide the ground level animal model data required for further clinical testing in humans. In summary, our system will extend our understanding of other respiratory microbes under smoking conditions or other co-morbidities, such as diabetes, to help guide clinicians to achieve better treatment outcomes.

## Data availability statement

The original contributions presented in the study are included in the article/supplementary material, further inquiries can be directed to the corresponding author/s.

## Ethics statement

The animal study was reviewed and approved by University of California, Riverside Institutional Animal Care and Use Committee (IACUC).

## Author contributions

JRC, WY, HD performed experiments. JRC and HD performed the CSE treatment. WY performed histology analysis. WY and JRC analyzed histology data. JC and DX performed and analysis the cytotoxicity analysis. JRC performed Statistics. JRC and RH wrote the draft of the manuscript and all authors contributed to the revising of the manuscript prior to submission. All authors contributed to the article and approved the submitted version.

## References

[1] Lee VJ Ho ZJM Goh EH Campbell H Cohen C Cozza V. Advances in measuring influenza burden of disease. Influenza Other Respir Viruses (2018) 12:3–9. doi: 10.1111/irv.12533 29460425 PMC5818353

[2] Anonymous. CDC Factsheet: Influenza disease burden (Atlanta, GA: Center for Disease Control and Prevention).

[3] Organization WH. WHO influenza seasonal factsheet 2020. (2020) (Geneva, Switzerland: World Health Organization).

[4] Yan SK Weycker D Sokolowski S. US Healthcare costs attributable to type a and type b influenza. Hum Vaccines Immunotherapeutics (2017) 13:2041–7. doi: 10.1080/21645515.2017.1345400 PMC5612050 28700268

[5] Bank W. GDP (current US$). Available at: https://data.worldbank.org/indicator/NY.GDP.MKTP.CD (Accessed 8/31/22).

[6] CDC. 2000-2001 INFLUENZA SEASON SUMMARY (2001). Available at: https://www.cdc.gov/flu/weekly/weeklyarchives2000-2001/00-01summary.htm (Accessed 8/22/22).

[7] CDC. 2001-02 INFLUENZA SEASON SUMMARY (2002). Available at: https://www.cdc.gov/flu/weekly/weeklyarchives2001-2002/01-02summary.htm (Accessed 8/22/22).

[8] CDC. 03 U.S. INFLUENZA SEASON SUMMARY (2002). Available at: https://www.cdc.gov/flu/weekly/weeklyarchives2002-2003/02-03summary.htm (Accessed 8/22/22).

[9] CDC. 2003 - 04 U.S. INFLUENZA SEASON SUMMARY (2004). Available at: https://www.cdc.gov/flu/weekly/weeklyarchives2003-2004/03-04summary.htm (Accessed 8/22/22).

[10] CDC. 2004-05 U.S. INFLUENZA SEASON SUMMARY (2005). Available at: https://www.cdc.gov/flu/weekly/weeklyarchives2004-2005/04-05summary.htm (Accessed 8/22/22).

[11] CDC. 2005-06 U.S. INFLUENZA SEASON SUMMARY (2006). Available at: https://www.cdc.gov/flu/weekly/weeklyarchives2005-2006/05-06summary.htm.

[12] CDC. 2006-07 U.S. INFLUENZA SEASON SUMMARY (2007). Available at: https://www.cdc.gov/flu/weekly/weeklyarchives2006-2007/06-07summary.htm.

[13] CDC. 2007-08 U.S. INFLUENZA SEASON SUMMARY (2008). Available at: https://www.cdc.gov/flu/weekly/weeklyarchives2007-2008/07-08summary.htm (Accessed 8/22/22).

[14] CDC. 2008-2009 influenza season summary (2009). Available at: https://www.cdc.gov/flu/weekly/weeklyarchives2008-2009/08-09summary.htm (Accessed 8/22/22).

[15] CDC. 2009-2010 influenza season summary (2010). Available at: https://www.cdc.gov/flu/weekly/weeklyarchives2009-2010/09-10Summary.htm (Accessed 8/22/22).

[16] CDC. Update: Influenza activity - united states, 2010-11 season, and composition of the 2011-12 influenza vaccine (2011). Available at: https://www.cdc.gov/mmwr/preview/mmwrhtml/mm6021a5.htm (Accessed 8/22/22). 21637185

[17] CDC. Update: Influenza activity - united states, 2011-12 season and composition of the 2012-13 influenza vaccine (2012). Available at: https://www.cdc.gov/mmwr/preview/mmwrhtml/mm6122a4.htm (Accessed 8/22/22). 22672977

[18] CDC. Influenza activity - united states, 2012-13 season and composition of the 2013-14 influenza vaccine (2013). Available at: https://www.cdc.gov/mmwr/preview/mmwrhtml/mm6223a5.htm?s_cid=mm6223a5_e (Accessed 8/22/22). PMC4604847 23760189

[19] CDC. Influenza activity - united states, 2013-14 season and composition of the 2014-15 influenza vaccines (2014). Available at: https://www.cdc.gov/mmwr/preview/mmwrhtml/mm6322a2.htm (Accessed 8/22/22). PMC5779357 24898165

[20] CDC. Influenza activity - united states, 2014-15 season and composition of the 2015-16 influenza vaccine (2015). Available at: https://www.cdc.gov/mmwr/preview/mmwrhtml/mm6421a5.htm (Accessed 8/22/22). PMC4584770 26042650

[21] CDC. Influenza activity - united states, 2015-16 season and composition of the 2016-17 influenza vaccine (2016). Available at: https://www.cdc.gov/mmwr/volumes/65/wr/mm6522a3.htm (Accessed 8/22/22). 10.15585/mmwr.mm6522a3 27281364

[22] CDC. Update: Influenza activity in the united states during the 2016–17 season and composition of the 2017–18 influenza vaccine (2017). Available at: https://www.cdc.gov/mmwr/volumes/66/wr/mm6625a3.htm (Accessed 8/22/22).

[23] CDC. Update: Influenza activity in the united states during the 2017–18 season and composition of the 2018–19 influenza vaccine (2018). Available at: https://www.cdc.gov/mmwr/volumes/67/wr/mm6722a4.htm?s_cid=mm6722a4_w (Accessed 8/22/22). 10.15585/mmwr.mm6722a4 PMC5991814 29879098

[24] CDC. FluView interactive map (2019-2020, weeks 40-26). Available at: https://gis.cdc.gov/grasp/fluview/fluportaldashboard.html (Accessed 8/22/22).

[25] Anonymous. Influenza in Europe, summary of the season 2017–18 (Solna Municipality, Sweden: European Centre for Disease Prevention and Control) (2018).

[26] Bhat YR. Influenza b infections in children: A review. World J Clin Pediatr (2020) 9:44–52. doi: 10.5409/wjcp.v9.i3.44 33442534 PMC7769779

[27] Anonymous. CDC Smoking & tobacco use factsheet. (2020) (Atlanta, GA: Center for Disease Control and Prevention).

[28] Control CfD. CDC Smoking & tobacco use: Health effects factsheet (Atlanta, GA: Center for Disease Control and Prevention) (2020).

[29] Almirall J Gonzalez CA Balanzo X Bolibar I. Proportion of community-acquired pneumonia cases attributable to tobacco smoking. Chest (1999) 116:375–9. doi: 10.1378/chest.116.2.375 10453865

[30] Almirall J Bolibar I Balanzo X Gonzalez CA. Risk factors for community-acquired pneumonia in adults: a population-based case-control study. Eur Respir J (1999) 13:349–55. doi: 10.1183/09031936.99.13234999 10065680

[31] Aryanpur M Masjedi MR Hosseini M Mortaz E Tabarsi P Soori H. Cigarette smoking in patients newly diagnosed with pulmonary tuberculosis in Iran. Int J Tuberculosis Lung Dis (2016) 20:679–84. doi: 10.5588/ijtld.15.0662 27084824

[32] Alcaide J Altet MN Plans P Parron I Folguera L Salto E. Cigarette smoking as a risk factor for tuberculosis in young adults: A case-control study. Tubercle Lung Dis (1996) 77:112–6. doi: 10.1016/S0962-8479(96)90024-6 8762844

[33] Smith GS Van Den Eeden SK Baxter R Shan J Van Rie A Herring AH. Cigarette smoking and pulmonary tuberculosis in northern California. J Epidemiol Community Health (2015) 69:568–73. doi: 10.1136/jech-2014-204292 25605864

[34] Bonacci RA Cruz-Hervert LP Garcia-Garcia L Reynales-Shigematsu LM Ferreyra-Reyes L Bobadilla-del-Valle M. Impact of cigarette smoking on rates and clinical prognosis of pulmonary tuberculosis in southern Mexico. J Infect (2013) 66:303–12. doi: 10.1016/j.jinf.2012.09.005 PMC3543482 22982014

[35] Pavic I Jurkovic M Pastar Z. Risk factors for acute respiratory tract infections in children. Collegium Antropologicum (2012) 36:539–42. 22856242

[36] Mazarico E Gomez-Roig MD Guirado L Lorente N Gonzalez-Bosquet E. Relationship between smoking, HPV infection, and risk of cervical cancer. Eur J Gynaecological Oncol (2015) 36:677–80. doi: 10.12892/ejgo2713.2015 26775350

[37] Kark JD Lebiush M Rannon L. Cigarette-smoking as a risk factor for epidemic a(H1n1) influenza in young men. N Engl J Med (1982) 307:1042–6. doi: 10.1056/NEJM198210213071702 7121513

[38] Finklea JF Sandifer SH Smith DD. Cigarette smoking and epidemic influenza. Am J Epidemiol (1969) 90:390. doi: 10.1093/oxfordjournals.aje.a121084 5356947

[39] Aronson MD Weiss ST Ben RL Komaroff AL. Association between cigarette smoking and acute respiratory tract illness in young adults. JAMA (1982) 248:181–3. doi: 10.1001/jama.1982.03330020025023 7087108

[40] Organization WH. Smoking and COVID-19 (Geneva, Switzerland: World Health Organization) (2020).

[41] Poudel R Daniels LB DeFilippis AP Hamburg NM Khan Y Keith RJ. Smoking is associated with increased risk of cardiovascular events, disease severity, and mortality among patients hospitalized for SARS-CoV-2 infections. PloS One (2022) 17:e0270763. doi: 10.1371/journal.pone.0270763 35839264 PMC9286231

[42] Gualano RC Hansen MJ Vlahos R Jones JE Park-Jones RA Deliyannis G. Cigarette smoke worsens lung inflammation and impairs resolution of influenza infection in mice. Respir Res (2008) 9:53. doi: 10.1186/1465-9921-9-53 18627612 PMC2483272

[43] Feng Y Kong Y Barnes PF Huang FF Klucar P Wang XS. Exposure to cigarette smoke inhibits the pulmonary T-cell response to influenza virus and mycobacterium tuberculosis. Infection Immun (2011) 79:229–37. doi: 10.1128/IAI.00709-10 PMC3019896 20974820

[44] Han Y Ling MT Mao HW Zheng J Liu M Lam KT. Influenza virus-induced lung inflammation was modulated by cigarette smoke exposure in mice. PLoS One (2014) 9:e86166. doi: 10.1371/journal.pone.0086166 24465940 PMC3897646

[45] Hong MJ Gu BH Madison MC Landers C Tung HY Kim M. Protective role of gamma delta T cells in cigarette smoke and influenza infection. Mucosal Immunol (2018) 11:894–908. doi: 10.1038/mi.2017.93 29091081 PMC5930147

[46] Kang MJ Lee CG Lee JY Dela Cruz CS Chen ZJ Enelow R. Cigarette smoke selectively enhances viral PAMP- and virus-induced pulmonary innate immune and remodeling responses in mice. J Clin Invest (2008) 118:2771–84. doi: 10.1172/JCI32709 PMC2483678 18654661

[47] Ferrero MR Garcia CC Dutra de Almeida M Torres Braz da Silva J Bianchi Reis Insuela D Teixeira Ferreira TP. CCR5 antagonist maraviroc inhibits acute exacerbation of lung inflammation triggered by influenza virus in cigarette smoke-exposed mice. Pharm (Basel) (2021) 14:A4552. doi: 10.3390/ph14070620 PMC8308708 34203121

[48] Robbins CS Bauer CMT Vujicic N Gaschler GJ Lichty BD Brown EG. Cigarette smoke impacts immune inflammatory responses to influenza in mice. Am J Respir Crit Care Med (2006) 174:1342–51. doi: 10.1164/rccm.200604-561OC 17023734

[49] Bauer CMT Zavitz CCJ Botelho FM Lambert KN Brown EG Mossman KL. Treating viral exacerbations of chronic obstructive pulmonary disease: Insights from a mouse model of cigarette smoke and H1N1 influenza infection. PloS One (2010) 5:e13251. doi: 10.1371/journal.pone.0013251 20967263 PMC2953496

[50] Wang JM Li QH Xie JG Xu YJ. Cigarette smoke inhibits BAFF expression and mucosal immunoglobulin a responses in the lung during influenza virus infection. Respir Res (2015) 16:37. doi: 10.1186/s12931-015-0201-y 25849069 PMC4364338

[51] Mebratu YA Smith KR Agga GE Tesfaigzi Y. Inflammation and emphysema in cigarette smoke-exposed mice when instilled with poly (I:C) or infected with influenza a or respiratory syncytial viruses. Respir Res (2016) 17:75. doi: 10.1186/s12931-016-0392-x 27363862 PMC4929744

[52] Ladomenou F Kafatos A Galanakis E. Environmental tobacco smoke exposure as a risk factor for infections in infancy. Acta Paediatrica (2009) 98:1137–41. doi: 10.1111/j.1651-2227.2009.01276.x 19302093

[53] Jedrychowski W Flak E. Maternal smoking during pregnancy and postnatal exposure to environmental tobacco smoke as predisposition factors to acute respiratory infections. Environ Health Perspect (1997) 105:302–6. doi: 10.1289/ehp.97105302 PMC1470010 9171991

[54] Miyahara R Takahashi K Anh NTH Thiem VD Suzuki M Yoshino H. Exposure to paternal tobacco smoking increased child hospitalization for lower respiratory infections but not for other diseases in Vietnam. Sci Rep (2017) 7:45481. doi: 10.1038/srep45481 28361961 PMC5374438

[55] Aedo G Miranda M Chavez MN Allende ML Egana JT. A reliable preclinical model to study the impact of cigarette smoke in development and disease. Curr Protoc Toxicol (2019) 80:e78. doi: 10.1002/cptx.78 31058471

[56] Elliott MK Sisson JH West WW Wyatt TA. Differential *in vivo* effects of whole cigarette smoke exposure versus cigarette smoke extract on mouse ciliated tracheal epithelium. Exp Lung Res (2006) 32:99–118. doi: 10.1080/01902140600710546 16754475 PMC2092449

[57] Duffney PF Embong AK McGuire CC Thatcher TH Phipps RP Sime PJ. Cigarette smoke increases susceptibility to infection in lung epithelial cells by upregulating caveolin-dependent endocytosis. PLoS One (2020) 15:e0232102. doi: 10.1371/journal.pone.0232102 32437367 PMC7241776

[58] Jaspers I Horvath KM Zhang WL Brighton LE Carson JL Noah TL. Reduced expression of IRF7 in nasal epithelial cells from smokers after infection with influenza. Am J Respir Cell Mol Biol (2010) 43:368–75. doi: 10.1165/rcmb.2009-0254OC PMC2933552 19880818

[59] Noah TL Zhou HB Monaco J Horvath K Herbst M Jaspers I. Tobacco smoke exposure and altered nasal responses to live attenuated influenza virus. Environ Health Perspect (2011) 119:78–83. doi: 10.1289/ehp.1002258 20920950 PMC3018504

[60] Health NIo. Dictionary of cancer terms: Mainstream smoke (Bethesda, Maryland: National Institute of Health).

[61] Health NIo. Secondhand smoke and cancer (Bethesda, Maryland: National Institute of Health).

[62] Jacob P 3rd Benowitz NL Destaillats H Gundel L Hang B Martins-Green M. Thirdhand smoke: New evidence, challenges, and future directions. Chem Res Toxicol (2017) 30:270–94. doi: 10.1021/acs.chemrestox.6b00343 PMC5501723 28001376

[63] Ghorani V Boskabady MH Khazdair MR Kianmeher M. Experimental animal models for COPD: A methodological review. Tob Induc Dis (2017) 15:25. doi: 10.1186/s12971-017-0130-2 28469539 PMC5414171

[64] Chen H Vlahos R Bozinovski S Jones J Anderson GP Morris MJ. Effect of short-term cigarette smoke exposure on body weight, appetite and brain neuropeptide y in mice. Neuropsychopharmacology (2005) 30:713–9. doi: 10.1038/sj.npp.1300597 15508020

[65] Chen H Hansen MJ Jones JE Vlahos R Bozinovski S Anderson GP. Cigarette smoke exposure reprograms the hypothalamic neuropeptide y axis to promote weight loss. Am J Respir Crit Care Med (2006) 173:1248–54. doi: 10.1164/rccm.200506-977OC 16531608

[66] Chavez J Hai R. Effects of cigarette smoking on influenza Virus/Host interplay. Pathogens (2021) 10:1636. doi: 10.3390/pathogens10121636 34959590 PMC8704216

[67] Boehme SA Franz-Bacon K Ludka J DiTirro DN Ly TW Bacon KB. MAP3K19 is overexpressed in COPD and is a central mediator of cigarette smoke-induced pulmonary inflammation and lower airway destruction. PLoS One (2016) 11:e0167169. doi: 10.1371/journal.pone.0167169 27935962 PMC5147866

[68] Goel R Bitzer Z Reilly SM Trushin N Foulds J Muscat J. Variation in free radical yields from US marketed cigarettes. Chem Res Toxicol (2017) 30:1038–45. doi: 10.1021/acs.chemrestox.6b00359 PMC5539526 28269983

[69] Machlin LJ Bendich A. FREE-RADICAL TISSUE-DAMAGE - PROTECTIVE ROLE OF ANTIOXIDANT NUTRIENTS. FASEB J (1987) 1:441–5. doi: 10.1096/fasebj.1.6.3315807 3315807

[70] Anonymous. National cancer institute: Harms of cigarette smoking and health benefits of quitting factsheet. Available at: https://www.cancer.gov/about-cancer/causes-prevention/risk/tobacco/cessation-fact-sheet#:~:text=Of%20the%20more%20than%207%2C000,least%2069%20can%20cause%20cancer.

[71] Maraskovsky E Chen WF Shortman K. IL-2 AND IFN-GAMMA ARE 2 NECESSARY LYMPHOKINES IN THE DEVELOPMENT OF CYTOLYTIC T-CELLS. J Immunol (1989) 143:1210–4. doi: 10.4049/jimmunol.143.4.1210 2501391

[72] Curtsinger JM Agarwal P Lins DC Mescher MF. Autocrine IFN-gamma promotes naive CD8 T cell differentiation and synergizes with IFN-alpha to stimulate strong function. J Immunol (2012) 189:659–68. doi: 10.4049/jimmunol.1102727 PMC3392455 22706089

[73] Castro F Cardoso AP Goncalves RM Serre K Oliveira MJ. Interferon-gamma at the crossroads of tumor immune surveillance or evasion. Front Immunol (2018) 9. doi: 10.3389/fimmu.2018.00847 PMC5945880 29780381

